# Long-Term Effects of AposTherapy in Patients with Osteoarthritis of the Knee: A Two-Year Followup

**DOI:** 10.1155/2013/689236

**Published:** 2013-03-03

**Authors:** Yaron Bar-Ziv, Eytan M. Debbi, Yuval Ran, Shaike Benedict, Nahum Halperin, Yiftah Beer

**Affiliations:** ^1^Department of Orthopedic Surgery, Assaf Harofeh Medical Center, Zerifin, Israel; ^2^Biorobotics and Biomechanics Lab, Faculty of Mechanical Engineering, Technion-Israel Institute of Technology, Haifa, Israel

## Abstract

Several biomechanics treatments for knee osteoarthritis (OA) have emerged with the goal of reducing pain and improving function. Through this, researchers have hoped to achieve a transition from the pathological gait patterns to coordinated motor responses. The purpose of the study was to determine the long-term effects of a therapy using a biomechanical device in patients with knee OA. Patients with knee OA were enrolled to active and control groups. The biomechanical device used in therapy (AposTherapy) was individually calibrated to each patient in the active group. Patients in the control group received standard treatment. Outcomes were the Western Ontario and McMaster Osteoarthritis Index (WOMAC), Aggregated Locomotor Function (ALF), Short Form 36 (SF-36), and Knee Society Score assessments. The active and control groups were similar at the baseline (group difference in all scores *P* > 0.05). The active group showed a larger improvement over time between groups in all three WOMAC categories (*F* = 16.8, 21.7, and 18.1 for pain, stiffness, and function; all *P* < 0.001), SF-36 Physical Scale (*F* = 5.8; *P* = 0.02), Knee Society Knee Score (*F* = 4.3; *P* = 0.044 ), and Knee Society Function Score (*F* = 6.5; *P* = 0.014 ). At the two-year endpoint, the active group showed significantly better results (all *P* ≤ 0.001). The groups showed a difference of 4.9, 5.6, and 4.7 for the WOMAC pain, stiffness, and function scores, respectively, 10.8 s in ALF score, 30.5 in SF-36 Physical Scale, 16.9 in SF-36 Mental Scale, 17.8 in Knee Society Knee Score, and 25.2 in Knee Society Function Score. The biomechanical therapy examined was shown to significantly reduce pain and improve function and quality of life of patients with knee OA over the long term.

## 1. Introduction

 Knee osteoarthritis (OA) is one of the leading causes of disability in the elderly [1]. Currently, there is no cure for knee OA, and therefore, the primary goal of treatment is to reduce pain and improve function [2]. In recent years, there has been growing evidence on the importance of biomechanical factors in knee OA. Several biomechanical treatments for knee OA have emerged with the goal of reducing pain and improving function. These treatments aim to unload the diseased articular surface by using wedged insoles, foot orthoses, or valgus braces [3–5]. Other treatments have instead aimed to modify neuromuscular patterns, with a specific goal of improving gait patterns. 

The knee adduction moment (KAM) is an important parameter of gait that has been examined in recent years. A varus alignment of the femur and tibia compresses the medial compartment of the knee [6]. KAM results from the medially directed vector of the ground reaction force (GRF) relative to the knee during the stance phase of gait, which creates greater compressive loads on the medial compartment relative to the lateral compartment [7, 8]. Patients with knee OA have a higher KAM relative to the normal population, which is believed to drive the rapid progression of the disease [9, 10].

By improving gait patterns, such as KAM, researchers have hoped to achieve a transition from the pathological gait patterns that characterize knee OA gait to coordinated motor responses [11]. This would require patients to undergo a process of motor learning. In order to meet the requirements for motor learning, these methods must incorporate challenges for the motor system in a graded and controlled fashion, with multiple repetitions within a functional context [12]. For example, a study by Barrios et al. [13] was able to improve gait patterns and reduce KAM in individuals with valgus knees through repetitive training on a treadmill [13]. In addition, research has also shown that motor learning can be accomplished under perturbation in closed kinematic chain movement in which the whole limb, rather than just a single joint, is regarded as a kinetic functional unit [11]. 

**Figure 1:**
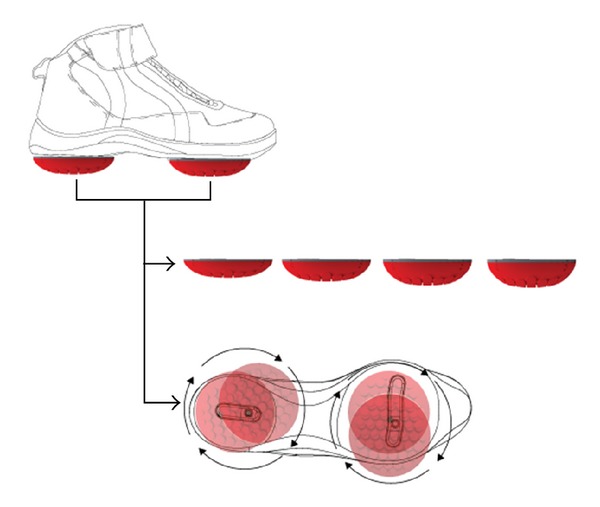
Biomechanical device used in therapy. The biomechanical device is comprised of four biomechanical elastic convex elements, with two attached under each foot using a foot-worn platform. The elements are attached under the hindfoot and forefoot regions using two mounting rails that allow for flexible positioning of each element under each region.

One biomechanical therapy (AposTherapy) that has received attention in recent years is thought to both unload the diseased articular surface as well as improve neuromuscular control. This therapy utilizes a unique biomechanical system (Apos system, Figure 1) that consists of two convex-shaped rubber elements attached to each of the patient's feet. One element is located under the hindfoot region, and one is located under the forefoot region of each foot. The elements are attached to the patient's foot on mounting rails embedded within the sole of a foot-worn device. The mounting rails enable flexible positioning of each element under each region. The elements are calibrated to the individual patient according to the pathology and motion characteristics.

Several studies by Haim et al. have examined the mechanism of the AposTherapy device and have shown that modifying the position of the biomechanical elements will precisely shift the center of pressure (COP) of each foot to a new location during gait [14, 15]. The COP can be shifted such that there are less external moments acting on the diseased articular surface. In addition, the convex shape of the elements puts the subject in a state of perturbation [16]. By having the subject walk with device everyday, the therapy is thereby thought to induce motor learning towards the desired neuromuscular gait pattern. Other studies have examined the effects of therapy on gait patterns and have shown that, over time, the therapy can be used to improve gait patterns, including KAM, in healthy individuals and in patients with knee OA [15, 17, 18]. Additional studies have shown that the biomechanical therapy is also able to modify the activation of lower limb muscles in healthy individuals and patients with knee OA as measured by electromyography [19, 20]. This signifies that the lower limb musculature of the subjects also adapts in a unique way to training.

In a previous controlled study [21], we examined the ability of this therapy to improve clinical symptoms of patients with knee OA after 8 weeks of therapy. Our results showed a significant improvement over time in pain, function, and quality of life in the active group compared to the control group after 8 weeks of therapy. The former study was limited by the short-term follow-up period. The purpose of the present study was to examine the effect of this biomechanical therapy on the level of pain, function, and quality of life in patients with knee OA over the course of 2 years.

## 2. Methods

### 2.1. Participants

The protocol was approved by the Institutional Helsinki Committee Registry of Assaf Harofeh Medical Center (Helsinki registration number 44/05 and NIH clinical trial registration number NCT00457132). All patients gave written informed consent prior to entering the study. Patients were recruited from the Department of Orthopedics of Assaf Harofeh Medical Center, Zerifin, Israel. Inclusion criteria were (1) symptomatic bilateral knee OA of the medial knee compartment for at least six months; (2) qualification of OA of the knee according to the American College of Rheumatology clinical criteria for OA of the knee, which include knee pain with at least 3 of the following: age > 50 years, stiffness < 30 minutes, crepitus, bony tenderness, bony enlargement, no palpable warmth [22]; (3) radiographically assessed OA of the knee according to the Kellgren & Lawrence (K&L) scale [23]. The K&L scale grades the knee according to one of four grades of severity, with I being the least severe and IV being the most severe OA. Only patients of grade II or above were included in the study. 

All patients had a varus knee alignment. Exclusion criteria were acute septic arthritis, inflammatory arthritis, patients with a history of increased tendency to fall, patients with a history of knee buckling, lack of physical or mental ability to perform or comply with the treatment procedure, diabetes mellitus, patients with a history of pathological osteoporotic fractures, symptomatic degenerative arthritis in lower limbs joints other than the knees (spine, hip, and ankle), severe back pain, and a history of lower limb orthopedic surgery other than knee arthroscopy. 

**Figure 2:**
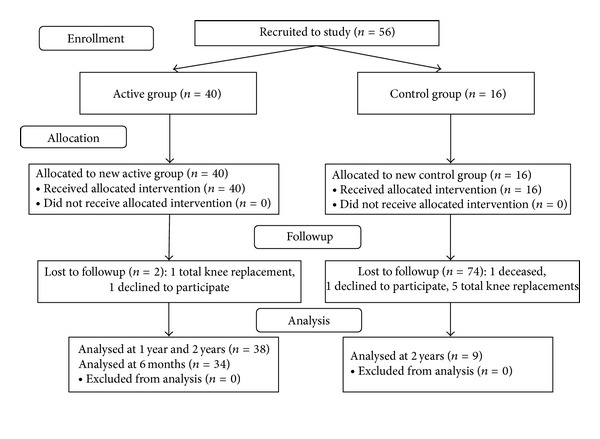
Flow chart of assessment, enrollment and followup.

Patients were recruited to the study by the senior orthopedic surgeon (N.H.) according to the inclusion and exclusion criteria. Patients in the active group received the biomechanical therapy, whereas patients in the control group received the same shoe, but without the biomechanical elements (walking on flat shoes like any other shoe). The active group was asked to return for a follow-up exam at six months, one year, and two years. The control group was only asked to return at two years for a followup. Some patients were lost to followup in both groups, and three patients did not arrive for the six-month follow-up in the active group (Figure 2). 

### 2.2. Intervention

The biomechanical therapy used for the present study is designed to combine COP manipulation in the foot with perturbation during walking. The therapy combines a biomechanical system with a specific treatment methodology (AposTherapy, Apos-Medical and Sports Technologies Ltd. Herzliya, Israel; Figure 2). The system consists of two convex-shaped biomechanical elements attached to each of the patient's feet (i.e., 4 elements total). One is located under the hindfoot region, and one is located under the forefoot region of each foot. The elements are attached to the patient's foot on mounting rails embedded within the sole of a shoe. The mounting rails enable flexible positioning of each element under each region.

The device can be calibrated to the individual patient according to the pathology and motion characteristics. Specifically in the case of medial compartment knee OA, the element under the hindfoot is shifted laterally from the baseline position. This shifts the COP in the foot laterally, thereby reducing the magnitude of the KAM acting on the knee joint [14, 24]. The element under the forefoot is shifted medially from the baseline. Both elements are moved until the patient reports minimal pain during walking. In addition, the convex nature of the elements puts the patient under constant perturbation. The device is calibrated by a physical therapist certified in the AposTherapy methodology. In the second phase of the therapy, the patient walks with the device for a prescribed amount of time, allowing for the biomechanical perturbations to be applied throughout all phases of gait in repetition. Perturbation is achieved through the controlled instability created when walking on two convex-shaped elements.

### 2.3. Active Group

At the start of the study, the biomechanical device was calibrated to all patients in the active group. The device was recalibrated, as necessary, at each followup and between followups. Once the device was calibrated, the patient was sent home with the device and was requested to train with the device by walking with it during his daily routine for a specified amount of time each day. The patient was told to begin with ten minutes of indoor walking each day and gradually build up to thirty minutes of daily outdoor walking by three months. Compliance to the therapy was maintained with a log as well as with follow-up phone calls. Individuals allocated to the control and research group were allowed to consume other treatment modalities as they saw necessary to treat their knee. Patients were monitored for any invasive treatments applied during this period. Furthermore, patients were instructed not to participate in other studies.

### 2.4. Control Group

The patients in the control group received the same walking shoe, but without the biomechanical elements. The patients were told to begin with ten minutes of indoor walking each day and gradually build up to thirty minutes of daily outdoor walking by three months. Compliance to the therapy was maintained with a log as well as with follow-up phone calls. Patients returned at two years for reevaluation. Individuals in the control group were allowed to undergo any other medical or physical therapy, as well as use pain medication, as they saw necessary to treat their knee. 

### 2.5. Outcome Measures

The primary outcomes of the study included the Western Ontario and McMaster Osteoarthritis Index (WOMAC) [25] and the Aggregated Locomotor Function (ALF) test [26]. The WOMAC contains 24 visual analogue scale (VAS) questions and is divided into three categories of pain, stiffness, and function. These scales are scored from 0 cm to 10 cm, with 0 = no symptoms and 10 = worst symptoms. Since the study population was made up of patients with bilateral knee OA, patients were requested to score the WOMAC for their most painful knee. The ALF score is a sum of the mean time (seconds) taken to complete three locomotor tasks while barefoot: walking eight meters, ascending and descending seven stairs, and transferring from a sitting to standing position. Each task was carried out separately with a break in between. The sum score was added up for all tasks. 

The secondary outcomes included the Short Form 36 (SF-36) health survey [27] and the Knee Society Score (KSS) [28]. The SF-36 is divided into eight categories: physical functioning, role limitation due to physical health, role limitation due to emotional health, energy/fatigue, emotional well-being, social functioning, pain, and general health. The physical component summary (PCS) and mental component summary (MCS) are summary scales of the eight categories. These scales are scored from 0–100, with higher scores indicating better states of health and quality of life. The KSS is measured by the clinician and is divided into a knee score (KSS-K) and function score (KSS-F). The KSS-K evaluates the function of the knee alone, while the KSS-F evaluated the function of the knee during physical activity. 

### 2.6. Statistical Analysis

The analysis was performed using SPSS software version 19.0 (SPSS, Chicago). Data were presented by frequencies and percentages for categorical variables and by means and standard deviations for continuous variables. Mean differences between the groups were presented with 95% confidence intervals (CIs). 

The criterion for significance (alpha) was set at 0.050, and the test was two tailed. With the proposed sample size of 38 pairs of cases, the study had a power exceeding 99.9% to yield a statistically significant result. This computation assumed that the population from which the sample would be drawn would have a mean difference of 3.0 with a standard deviation of 2.0. The observed value was tested against a theoretical value (constant) of 0.00. With the same assumption, a sample size of 9 pairs of cases would have had power of 97.5% to yield a statistically significant result.

Kolmogorov-Smirnov tests of the study outcome distribution showed that all the results were normally distributed. An independent *t*-test (two tailed) was used to compare the patient characteristics of age at the baseline, as well as all the outcome measures at the baseline and after two years. The patient characteristics of sex and Kellgren and Lawrence radiographic knee OA (K&L) score between groups were compared at the baseline using a chi-square test. Changes within the groups and differences between the groups in all outcomes over time (time by treatment interaction) were measured using a repeated measures analysis of variance (ANOVA) test. The significant level was set to 0.05.

## 3. Results

A total of 56 patients (15 males, 41 females, age 65.1 (SD 7.9) years) were enrolled to the study. The active group was comprised of 40 patients (10 males, 30 females, age 64.1 (SD 7.5) years), and the control group was comprised of 16 patients (5 males, 11 females, age 67.4 (SD 8.6) years). At the two-year endpoint, 38 patients and 9 patients remained in each group, respectively (Figure 1). At the baseline, the groups were comparable in patient characteristics (Table 1), primary outcomes (Table 2), and secondary outcomes (Table 3).

**Figure 3:**
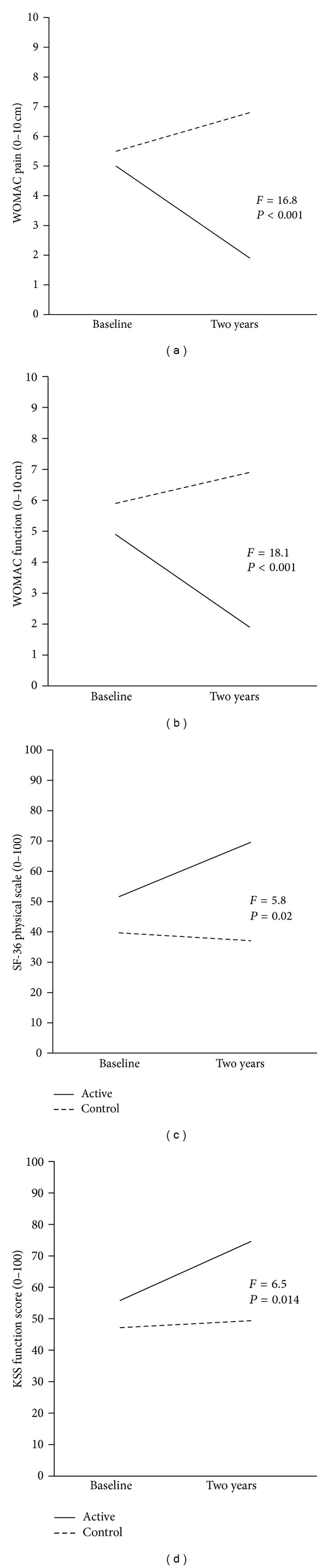
Time by treatment interaction graphs between groups over two years. There was a significant difference in improvement over time between groups in the Western Ontario and McMaster Osteoarthritis Index (WOMAC) for pain and function, the Short Form 36 (SF-36) physical component summary, and in the Knee Society Score (KSS) Function Scale.

**Figure 4:**
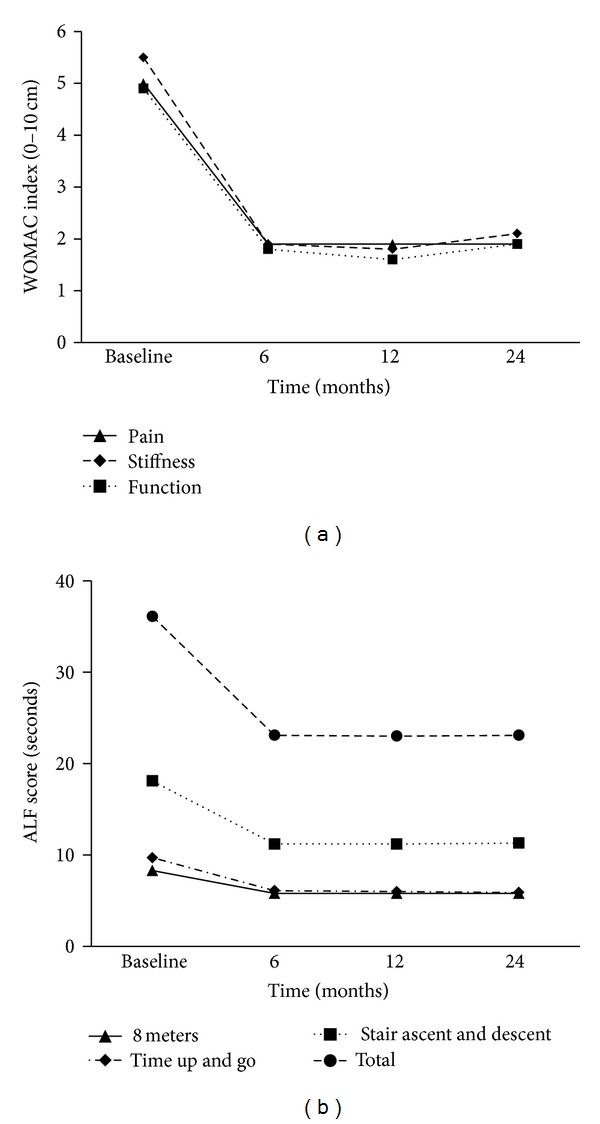
WOMAC and ALF scores over time in the experimental group. The analysis over time shows that the improvements in all three categories of the Western Ontario and McMaster Osteoarthritis Index (WOMAC) and in the Aggregated Locomotor Function Score (ALF) are maintained throughout the study.

A significant difference was found between the active and control groups in all three WOMAC categories (pain, stiffness, and function) at the two-year endpoint (all *P* < 0.001; Table 2). There was also a significant difference in improvement over time between groups in all three categories (*F* for interaction =16.8, 21.7 and 18.1 for pain, stiffness, and function, resp.; all *P* < 0.001). Figures 3(a) and 3(b) show the time by treatment interaction for WOMAC pain and WOMAC function, respectively. An analysis for the active group over time showed that the improvement in all three categories was maintained throughout the study (Figure 4(a)).

The improvements in pain and function in the WOMAC questionnaires qualified as a clinical response to treatment according to the Outcome Measures in Rheumatology Clinical Trials (OMERACT)-Osteoarthritis Research Society International (OARSI) set of responder criteria. These are an improvement in either pain or function of at least fifty percent with a decrease of 2.0 cm on a VAS or an improvement in both pain and function of at least twenty percent with a decrease of 1.0 cm on a VAS [29]. The results of pain and function in the WOMAC questionnaires in the present study meet both these clinical improvement criteria since the changes in pain and function were greater than 50% and greater than 2.0 cm on the VAS.

A significant difference between the active and control groups was also found in ALF score at the two-year endpoint (*P* < 0.001; Table 2). The two groups did not differ significantly in their improvement over time (*F* for interaction =0.67; *P* = 0.419; Figure 3(c)). The analysis of the active group over time showed that the improvement in ALF score in the active group was maintained throughout the study (Figure 4(b)).

At the two-year endpoint, a significant difference was found between groups in all categories of the SF-36 except for the category of emotional well-being. This is reflected in the two summary indices of the SF-36: the SF-36 PCS and SF-36 MCS (*P* < 0.001, *P* = 0.001, resp.; Table 3). There was a significant difference in improvement over time between groups in the SF-36 PCS (*F* for interaction =5.8; *P* = 0.02; Figure 3(c)) but not in the SF-36 MCS (*F* for interaction =0.032; *P* = 0.86). 

At the two-year endpoint, a significant difference was found between groups in the KSS-K and the KSS-F (*P* < 0.001, *P* = 0.001, resp.; Table 3). The two groups also differed significantly in their improvement over time in the KSS-K (*F* for interaction =4.3; *P* = 0.044) and the KSS-F (*F* for interaction =6.5; *P* = 0.014; Figure 3(d)). The analysis of the active group over time showed that the improvement in both outcomes was maintained for the rest of the study period.

## 4. Discussion

Patients treated with the biomechanical therapy showed greater improvements at the study endpoint in all the study outcomes, as well as greater improvement over time in most of the study outcomes. Interestingly, the results of the ALF test showed that the groups did not show a significant difference in improvement over time. This suggests that the control group may have improved in function over the two years, but not to the extent of the active group. The changes in function in the control group may be due to other therapies that this group used during the study period. Since the control group was not examined as often as the active group, another explanation may be that the control group did not improve in function, but rather that the study was not strong enough at finding the difference in improvement over time between groups.

The groups also differed in the number of total knee replacements (TKRs) performed after two years in each group. One patient from the active group required a TKR during the study period (2.6%), while 5 patients (31%) of the control group required a TKR during the two-year study period. The TKR was documented through our follow-up phone calls to the patients over time. The procedures were performed at various medical centers throughout the country where the individual was referred to surgery. The type of prosthetic was not documented for the purposes of the present study. 

The previous study showed that the improvements in the active group were still rising at the eight-week endpoint. The results of the present study at six months were only slightly higher than the results after eight weeks that were seen in the previous study. From six months to the end of the study, the improvements remained stable. This suggests that the majority of improvements with the biomechanical therapy are achieved within the first eight weeks of therapy. Furthermore, these improvements remain stable as long as treatment is maintained. 

The present study also supports the results of a previous study by Elbaz et al. [17] that also evaluated this biomechanical therapy. Their study showed that patients with knee OA treated with the therapy reported improvements in pain, function and quality of life as demonstrated by self-evaluation questionnaires [17]. Their study, however, evaluated patients over only twelve weeks of therapy and did not incorporate a control group.

Researchers have presented several theories explaining how this therapy may reduce pain and improve function in patients with OA of the knee. Several studies by Haim et al. showed that the device used in this therapy can unload the diseased articular surface of the joint with knee OA and thereby reduce pain. This was witnessed in the current study in that immediately after calibration patients reported diminished pain or no pain while using the biomechanical device. By reducing pain, the therapy gives the patients the ability to train without pain. Over time the therapy may allow the patient to regain strength, function, and lower pain levels.

The therapy may also reduce pain and improve function by educating the neuromuscular system of these patients to walk in a less pathological manner [17]. Motor learning in the human body is a complicated task and must be incorporated in a graded and controlled fashion, with multiple repetitions within a functional context [12]. Fitzgerald et al. [11] showed that this could be accomplished through perturbation during repetitive actions [11]. The therapy used in the present study uses COP manipulation to realign the limb towards a normal biomechanical alignment while minimizing any preexisting pain. By combining the changes in alignment with perturbation and repetition over time, the therapy may educate the neuromuscular system to acquire the ability to walk in the new alignment, which in turn allows the patient to walk in the new gait pattern even when the biomechanical device is removed.

As a therapy, the biomechanical intervention also showed high compliance over the course of two years. This may be due to the fact that the actual therapy was very easy to apply to patients. The patients also reported ease in using the device since they only had to wear the device while walking in their regular environment or while carrying out simple chores. 

There were several limitations to the present study. Firstly, in contrast to our previous study, the present study was unblended, and the two groups were not randomized. Nevertheless, the two groups were equal at the baseline in terms of patient characteristics and clinical outcomes. Secondly, due to the study logistics, the control group could only be asked to arrive for a follow-up exam at two year without evaluations before then. This limits our knowledge of how this group faired over time.

There are several ways in which the study could have been improved. The study could have attempted to discontinue treatment with the device to see if the improvements are maintained without therapy. This addition to the study could allow researchers to determine if and when the therapy can be terminated. This may test whether the patients acquired a new action that they will maintain on their own or whether the patients need continuous training to maintain their new gait patterns. The present study could also benefits from spatiotemporal, kinetic, and kinematic gait analyses of the patients over time when the treatment device is removed. This could help determine which, if any, changes in gait the body's motor learning system is able to acquire from therapy.

## 5. Conclusions

The present study shows that patients with knee OA treated with AposTherapy over time demonstrate a significant reduction in pain and a significant improvement in function and quality of life. These improvements peak after eight weeks of therapy and remain stable for two years as long as treatment is maintained. 

## Figures and Tables

**Table 1:** Baseline patient characteristics.

Characteristic	Active (*N* = 40)	Control (*N* = 16)	*P* value*
Age (mean age ± SD)	64.1 ± 7.5	67.4 ± 8.6	0.53
Females (%)	75	69	0.17
Kellgren and Lawrence (K&L)			
K&L Grade 2 (%)	17.5	18.8	
K&L Grade 3 (%)	25.0	31.2	0.77
K&L Grade 4 (%)	57.5	50.0	

K&L: Kellgren and Lawrence radiographic grading scale for knee osteoarthritis.

*P ≤ 0.05 was considered statistically significant.

Groups showed no significant differences at the baseline.

**Table 2:** Primary outcomes.

Outcome	Baseline	Mean difference ± SE (95% CI)	*P* value	2 years	Mean difference ± SE (95% CI)	*P* value	Time by treatment interaction (*F*; significance)
WOMAC pain							
Active	5.0 ± 2.8	−0.5 ± 1.0		1.9 ± 1.6	−4.9 ± 0.6		
Control	5.5 ± 3.3	(−2.6, 1.6)	0.7	6.8 ± 2.0	(−6.2, −3.7)	<0.001*	*F* = 16.8; *P* < 0.001
WOMAC stiffness							
Active	5.5 ± 3.1	−0.1 ± 1.2		2.1 ± 1.7	−5.6 ± 0.6		
Control	5.6 ± 3.3	(−2.4, 2.3)	0.9	7.7 ± 1.5	(−6.8, −4.3)	<0.001*	*F* = 21.7; *P* < 0.001
WOMAC function							
Active	4.9 ± 2.6	−0.9 ± 0.9		1.9 ± 1.3	−4.7 ± 0.5		
Control	5.9 ± 2.5	(−2.8, 1.0)	0.3	6.6 ± 1.7	(−5.7, −3.6)	<0.001*	*F* = 18.1; *P* < 0.001
ALF score							
Active	35.5 ± 10.3	−5.8 ± 5.0		23.1 ± 6.4	−10.8 ± 2.5		
Control	41.9 ± 22.3	(−2.8, 0.9)	0.3	33.9 ± 7.3	(−15.8, −5.8)	<0.001*	*F* = 0.67; *P* = 0.419

WOMAC: Western Ontario and McMaster Osteoarthritis Index; ALF: Aggregated Locomotor Function.

*P ≤ 0.05 was considered statistically significant. Groups showed no significant differences at the baseline. Groups showed significant difference in all outcomes after two years. There was a significant difference in improvement over time between groups in all outcomes except for the ALF.

**Table 3:** Secondary outcomes.

Outcome	Baseline	Mean difference ± SE (95% CI)	*P* value	2 years	Mean difference ± SE (95% CI)	*P* value	Time by treatment interaction (*F*; significance)
SF-36 PCS							
Active	51.9 ± 19.2	11.8 ± 7.0		67.6 ± 16.3	30.5 ± 5.6		
Control	39.7 ± 17.8	(−2.2, 25.8)	0.1	37.1 ± 14.9	(18.5, 42.6)	<0.001*	*F* = 5.8; *P* = 0.02
SF-36 MCS							
Active	64.7 ± 19.6	14.1 ± 7.2		73.7 ± 13.1	16.9 ± 4.8		
Control	50.3 ± 19.7	(−0.5, 28.6)	0.1	56.8 ± 12.5	(7.2, 26.6)	<0.001*	*F* = 0.032; *P* = 0.86
Knee Society Knee Score							
Active	58.1 ± 18.8	3.4 ± 6.8		77.8 ± 12.1	17.8 ± 4.7		
Control	54.1 ± 16.3	(−10.3, 17.2)	0.6	60.0 ± 14.9	(8.3, 27.3)	<0.001*	*F* = 4.3; *P* = 0.044
Knee Society Function Score							
Active	57.1 ± 16.0	8.5 ± 6.5		74.6 ± 18.3	25.2 ± 6.9		
Control	47.2 ± 16.4	(−4.6, 21.7)	0.2	49.4 ± 19.3	(11.2, 39.0)	<0.001*	*F* = 6.5; *P* = 0.014

SF-36: Short Form 36 (SF-36).

*P ≤ 0.05 was considered statistically significant. Groups showed no significant differences at the baseline. Groups showed significant differences in all outcomes after two years. There was a significant difference in improvement over time between groups in all outcomes except for the SF-36 mental component summary (MCS).
